# Molecular Characterization and Expression of Cytochrome P450 Aromatase in Atlantic Croaker Brain: Regulation by Antioxidant Status and Nitric Oxide Synthase During Hypoxia Stress

**DOI:** 10.3389/fphys.2021.720200

**Published:** 2021-08-09

**Authors:** Md Saydur Rahman, Peter Thomas

**Affiliations:** ^1^School of Earth, Environmental and Marine Sciences, University of Texas Rio Grande Valley, Brownsville, TX, United States; ^2^Marine Science Institute, University of Texas at Austin, Port Aransas, TX, United States

**Keywords:** brain, aromatase, antioxidant, fish, hypoxia

## Abstract

We have previously shown that nitric oxide synthase (NOS, an enzyme) is significantly increased during hypoxic stress in Atlantic croaker brains and modulated by an antioxidant (AOX). However, the influence of NOS and AOX on cytochrome P450 aromatase (AROM, CYP19a1, an enzyme) activity on vertebrate brains during hypoxic stress is largely unknown. In this study, we characterized brain AROM (bAROM, CYP19a1b) cDNA in croaker and examined the interactive effects of hypoxia and a NOS-inhibitor or AOX on AROM activity. The amino acid sequence of croaker bAROM cDNA is highly homologous (76–80%) to other marine teleost bAROM cDNAs. Both real-time PCR and Northern blot analyses showed that bAROM transcript (size: ∼2.8 kb) is highly expressed in the preoptic-anterior hypothalamus (POAH). Hypoxia exposure (dissolved oxygen, DO: 1.7 mg/L for 4 weeks) caused significant decreases in hypothalamic AROM activity, bAROM mRNA and protein expressions. Hypothalamic AROM activity and mRNA levels were also decreased by pharmacological treatment with *N*-ethylmaleimide (NEM, an alkylating drug that modifies sulfhydryl groups) of fish exposed to normoxic (DO: ∼6.5 mg/L) conditions. On the other hand, treatments with *N*ω-nitro-L-arginine methyl ester (NAME, a competitive NOS-inhibitor) or vitamin-E (Vit-E, a powerful AOX) prevented the downregulation of hypothalamic AROM activity and mRNA levels in hypoxic fish. Moreover, NAME and Vit-E treatments also restored gonadal growth in hypoxic fish. Double-labeled immunohistochemistry results showed that AROM and NOS proteins are co-expressed with NADPH oxidase (generates superoxide anion) in the POAH. Collectively, these results suggest that the hypoxia-induced downregulation of AROM activity in teleost brains is influenced by neuronal NOS activity and AOX status. The present study provides, to the best of our knowledge, the first evidence of restoration of AROM levels in vertebrate brains by a competitive NOS-inhibitor and potent AOX during hypoxic stress.

## Introduction

The teleost brain is a major target organ for investigating the molecular, cellular, physiological, neuroendocrine, and behavioral responses of fishes to environmental stressors (Zohar et al., 2009; Diotel et al., 2011). Importantly, like in other vertebrates, the teleost brain has higher oxygen utilization and metabolic rates than other organs such as the gills, gonads, kidney, and liver, and uses around 20% of the total oxygen consumed by the body (Lahiri et al., 2006). Thus, neuroendocrine and other brain functions are highly susceptible to reduced oxygen availability in teleost fishes when they are exposed to hypoxic environments (dissolved oxygen (DO) levels are <2.0 mg/L in marine environments; Diaz and Rosenberg, 2008), less than 1/3 the DO levels in normoxic environments (Wu, 2002; Thomas et al., 2007; Thomas and Rahman, 2009; Rahman and Thomas, 2017).

**FIGURE 1:**
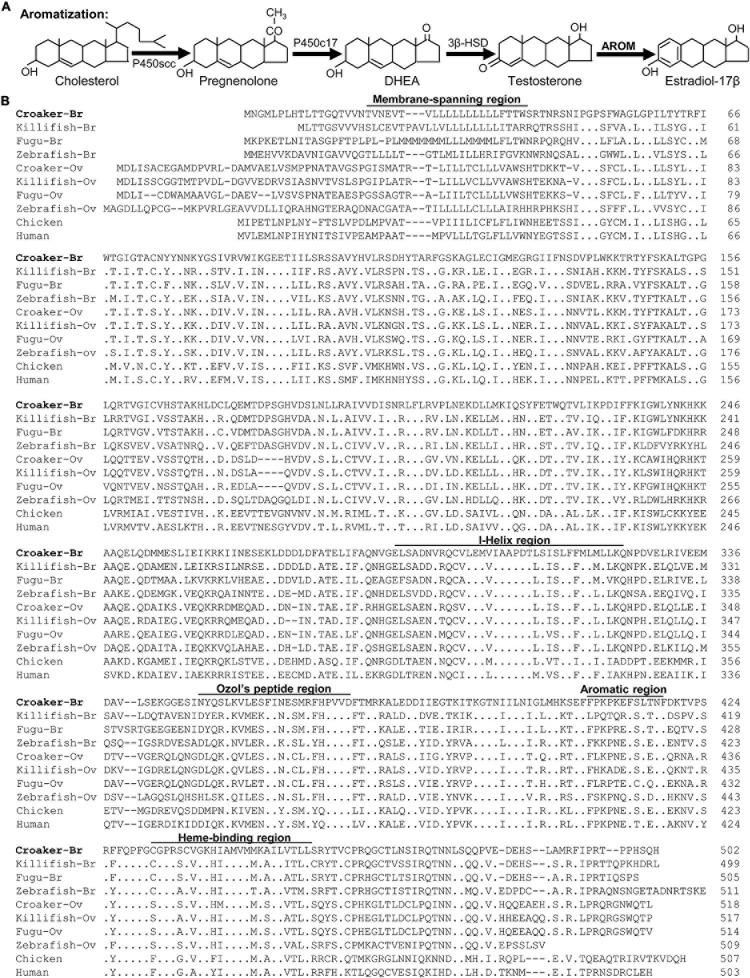
**(A)** Schematic diagram of estradiol -17β synthesis. Testosterone is synthesized from dehydroepiandrosterone (DHEA). Testosterone is then converted to estradiol -17β by the enzyme cytochrome P450 aromatase (AROM). **(B)** Alignment of the deduced amino acid sequences of membrane-spanning domain, I-Helix region, Ozol’s peptide region, aromatic and heme-binding domains of croaker brain AROM (bAROM) with the AROM-related proteins of other vertebrates. Dots indicate residues that are identical to croaker bAROM. Dashes indicate gaps introduced to facilitate alignment. GenBank accession numbers for the sequence of brain (Br) ovary (Ov) AROM proteins used are as follows: Atlantic croaker-Br (JF300170), killifish-Br (AY428666), fugu-Br (AB330137), zebrafish-Br (AAV41033), croaker-Ov (DQ184486), killifish-Ov (AY428665), fugu-OV (AB330136), zebrafish-Ov (O42145), chicken (P19098), and human (P11511).

The complex processes and series of events that occur during reproduction in vertebrates are mainly controlled by hormones secreted by the hypothalamus-pituitary-gonadal axis (Van Der Kraak, 2009; Fuzzen et al., 2011; Parhar et al., 2016; Acevedo-Rodriguez et al., 2018). The hypothalamus synthesizes gonadotropin-releasing hormone (GnRH), the primary neuropeptide controlling reproduction, neurotransmitters such as serotonin (5-HT) that regulate GnRH release, and also has high expression of cytochrome P450 aromatase (AROM, CYP19a1, a key enzyme for estradiol-17β synthesis that converts C19 androgens to C18 estrogens, Figure 1A; Piferrer and Blázquez, 2005; Page et al., 2010; Diotel et al., 2011; Balthazart and Ball, 2012). There is extensive evidence that AROM plays crucial roles in both reproductive and non-reproductive functions in vertebrates (Simpson and Davis, 2001; Lang et al., 2002; Garcia-Segura, 2008). Most tetrapods have a single AROM (CYP19a1) transcript in the brain, gonads, and other tissues, while teleost fishes have two distinct AROM transcripts: (i) an ovarian-type AROM (oAROM, CYP19a1a) transcript expressed mainly in the ovary, and (ii) a brain-type AROM (bAROM, CYP19a1b) transcript expressed mainly in the brain (Tchoudakova and Callard, 1998; Gonzalez and Piferrer, 2002, 2003; Piferrer and Blázquez, 2005; Diotel et al., 2011). Teleost hypothalami have especially high AROM activities and show sex differences in AROM expression with higher levels in females than in males (Pasmanik and Callard, 1985; Diotel et al., 2011; Okubo et al., 2011; Chaube et al., 2015). The maintenance of high AROM levels in teleost brains is thought to contribute to the maintaining of a high level of sexual plasticity in fishes into adulthood (Page et al., 2010; Okubo et al., 2011). The cellular expression of AROM in the teleost brain differs from the neuronal expression in mammals in that it is exclusively expressed in radial glial cells where it permits a high rate of neurogenesis throughout life (Page et al., 2010; Okubo et al., 2011). However, under pathological conditions AROM is also expressed in astrocyte glial cells in the brain of mammals, where it exerts protective functions such as neurogenesis (Garcia-Segura et al., 2003; Roselli, 2007). Thus, in addition to its reproductive neuroendocrine functions, bAROM also plays important roles in maintaining sexual plasticity and neuroprotection and therefore is a critical enzyme regulating brain function in teleost fishes.

Due to its essential role in neuroendocrine and other brain functions, there has been mounting concern that AROM activity and/or expression in the teleost brain may be disrupted during environmental exposure to hypoxia. Previous studies demonstrated that hypoxia inhibits reproductive neuroendocrine functions by interfering with AROM activity and expression and altering GnRH and 5-HT levels in the brain of vertebrates (Shang et al., 2006; Thomas et al., 2007; Kumar, 2011; Thomas and Rahman, 2012). Studies in teleost fishes have demonstrated that hypoxia decreases AROM activity in the brain and inhibits plasma estradiol-17β (E_2_) levels in zebrafish (Shang et al., 2006). Our recent field studies have shown that the marked inhibition of gonadal development (e.g., lower fecundity and sperm quantity) and masculinization of ovaries is associated with lower bAROM and GnRH mRNA levels in hypothalamic tissues, lower estrogen receptor alpha (ERα, an index of estrogen signaling) mRNA levels in the liver and reduced vitellogenin production, and decreased AROM mRNA levels in the ovaries of Atlantic croaker collected from hypoxic sites in the northern Gulf of Mexico, a second largest hypoxic zone (also called “dead zone”) in the world (Thomas and Rahman, 2012). Moreover, our laboratory studies have demonstrated that hypoxia inhibits hypothalamic GnRH and 5-HT levels and tryptophan hydroxylase (TPH, a rate limiting enzyme for 5-TH synthesis) activity in croaker (Thomas et al., 2007; Rahman and Thomas, 2009).

In addition to reproductive impairment, there is extensive evidence that hypoxia exposure causes a wide range of other deleterious effects such as behavioral, immunological, biochemical, and physiological modifications (Wu, 2002; Abdel-Tawwab et al., 2019). Many hypoxic effects are mediated through overproduction of reactive oxygen species (ROS, e.g., superoxide anion, O_2_^∙-^; hydrogen peroxide, H_2_O_2_; hydroxyl radical, ^⋅^OH) and reactive nitrogen species (RNS, e.g., nitric oxide, NO; peroxynitrite, ONOO^–^) through activation and/or expression of NADPH oxidase (NOX) and nitric oxide synthase (NOS) in the brains and peripheral tissues of vertebrates (Nikinmaa, 2002; Bedard and Krause, 2007). For example, hypoxia increases neuronal NOS (nNOS) activity, mRNA levels, and protein expression in porcine and rat brains (Yamamoto et al., 2003; Ward et al., 2005; Mishra et al., 2006; McLaren et al., 2007; Tsui et al., 2011). Moreover, a recent study has shown that acute exposure of rats to hypoxia caused a significant increase in brain NOS expression, which resulted in increased NO production as indicated by elevated brain levels of NO metabolites (NOx, nitrate and nitrite) and decreases in antioxidant defense systems such as reductions in glutathione levels (GSH) levels and GSH peroxidase, superoxide dismutase (SOD) activities, resulting in elevated apoptosis (Coimbra-Costa et al., 2017). Similarly, hypoxia induces the activity/expression of caspase-3, leading to an increase the number of apoptotic cells in the brain and ovary of teleost fishes (Lu et al., 2005; Ondricek and Thomas, 2018). Moreover, we have recently demonstrated that hypoxia increases nNOS mRNA and protein expression, and O_2_^∙-^ production in croaker brain (Rahman and Thomas, 2015). Hypoxia also stimulates eNOS protein expression and O_2_^∙-^ production in liver tissues and plasma NOx contents in croaker (Rahman and Thomas, 2011, 2012). Together, the studies previously described demonstrate that hypoxia stimulates NOS and NOX expression and activates free radicals (e.g., NO and O_2_^∙-^) production, thus increasing oxidative and nitrative stress, leading to increased cellular apoptosis, which results in impairment of brain functions in vertebrates.

We have extensively documented oxidative stress and increased nNOS mRNA and protein levels in the hypothalami of croaker exposed to hypoxia in controlled laboratory studies as well in the brains of croaker collected from hypoxic sites in the northern Gulf of Mexico dead zone (Rahman and Thomas, 2015, 2017). We have also shown that administration of estradiol-17β, vitamin-E, an antioxidant (AOX, a potent peroxyl radical scavenger; Traber and Atkinson, 2007), or a NOS-inhibitor increases TPH activity and 5-HT levels and decreases nNOS mRNA levels and O_2_^∙-^ production in croaker brains under hypoxic conditions (Rahman and Thomas, 2014, 2015). The results suggest that endogenous AROM, nNOS or NOX expressions, and/or antioxidant levels in the brain are essential for maintaining optimal reproductive neuroendocrine functions of teleost fishes. However, we are unaware of any *in vivo* studies to date that have examined the effects of antioxidants or NOS-inhibitors on AROM activity, or the roles of nNOS-NOX in AROM regulation in the vertebrate brain during hypoxia exposure. Therefore, the aims of this study were to determine whether chronic hypoxia exposure causes a decline in bAROM in croaker hypothalami and, in addition, if bAROM levels and activity are influenced by nNOS activity and antioxidant status under hypoxic conditions. First, croaker bAROM cDNA was cloned, characterized, and its expression pattern examined in peripheral tissues and discrete brain areas. Next, the effects of hypoxia exposure on AROM activity and bAROM mRNA and protein expressions in hypothalamic tissues and gonadal development in croaker were investigated. A major objective was to determine whether AROM expression and activity in hypoxia-exposed fish were restored by pharmacological treatments that inhibited nNOS activity and increased the antioxidant status or mimicked by treatment of normoxia-exposed fish with an alkylating drug. bAROM expression and activity were measured in hypothalami of croaker chronically treated with an alkylating drug, *N*-ethylmaleimide (NEM), which covalently modifies sulfhydryl groups to produce NO, and a NOS-inhibitor, *N*ω-nitro-L-arginine methyl ester, and an antioxidant, Vit-E regulation under either hypoxic or normoxic conditions. Finally, we investigated the co-localization of bAROM, nNOS, and NOX proteins in croaker hypothalami to determine their potential interactions.

## Materials and Methods

### Fish Collection and Acclimation

Young adult (year 1) Atlantic croaker (croaker, *Micropogonias undulatus*) were caught by shrimp trawl by a local fisherman near Port Aransas, TX, United States. The average length and body weight (BW) of croaker were 10–11 cm and 12–18 g, respectively. Fish were kept in aerated seawater, transported to the University of Texas Marine Science Institute campus, and treated with Paracide-F in seawater (170 ppm for 1 h) to diminish parasite infections. Fish were placed in large indoor tanks (4,727 L) and acclimated with recirculating seawater system (salinity: 30–32 ppt) at ambient seawater temperature (22 ± 1^*o*^C) and control photoperiod (11L:13D) for 3 months. Fish were fed chopped frozen shrimp daily (3% BW/day) during the acclimation period.

### Experiment 1: Effects of Hypoxia Exposure of Aromatase Expression in Croaker Brain

A detailed description of the hypoxia-exposure system used in this study has been reported previously (Supplementary Figure 1; Rahman and Thomas, 2009). Briefly, hypoxic conditions in the experimental tanks (capacity: 2,025 L including biofilter) were maintained by lowering the aeration gradually through an air-flow meter (SCFH AIR, Key Instruments, Trevose, PA, United States) and adjusting the dissolved oxygen (DO) level to ∼1.7 mg/L which was achieved within 2–3 days. Fish (30 mixed-sex fish/tank) were exposed in normoxic (DO: ∼6.5 mg/L) and hypoxic (DO: ∼1.7 mg/L) conditions for 4 weeks under control photoperiod (11L:13D) and temperature (22 ± 1^*o*^C) conditions. The exposure regimen used in this study based on that used in our previous hypoxia exposure experiments with croaker (Thomas et al., 2007; Rahman and Thomas, 2009, 2014, 2015). Fish were fed chopped frozen shrimp daily (3% BW/day) during the experimental period. At the end of the experiments, fish were euthanized using quinaldine (20 mg/L in seawater) and sacrificed under deep anesthesia. Brain tissues were carefully collected, placed in a histology cassette, submerged in ice-cold 4% paraformaldehyde (pH 7.4), and stored at 4^*o*^C prior to the preparation of immunohistochemical analysis. Brain tissues for protein and mRNA analyses were collected in 1.5 ml RNase/DNase-free microcentrifuge tubes, frozen in liquid nitrogen and stored at –80°C for later Western blot, radioenzymatic assay, and quantitative real-time PCR analyses.

### Experiment 2: Effects of Hypoxia and Pharmacological Treatments on Aromatase Regulation in Croaker Brain

A detailed description of fish collection, acclimatization and hypoxia-exposure system used in experiment 2 has been described previously (Rahman and Thomas, 2015). Briefly, for combined effects of hypoxia and pharmacological treatments, 30 mixed-sex fish were stocked in each of six experimental tanks under controlled laboratory conditions. After anesthetization, fish were given an intraperitoneal (i.p.) injection (1 μg/g BW) either with saline, *N*-ethylmaleimide (NEM, an alkylating agent that reacts with sulfhydryls; MilliporeSigma, Burlington, MA, United States), *N*ω-nitro-L-arginine methyl ester (NAME, a NOS-inhibitor; MilliporeSigma) or Vitamin-E (Vit-E, a powerful antioxidant, AOX; MilliporeSigma). The treatments (i.p. injection in each drug: 1 μg/g BW) were repeated every 4 days for 4 weeks. In the normoxia-exposed group, fish were injected with NEM, an alkylating agent, to determine whether it impaired hypothalamic aromatase expression similar to hypoxic fish and also with AOX treatment groups. In the hypoxia-exposed group, on the other hand, fish were treated with NAME, a NOS inhibitor, and AOX to determine if they recovered hypothalamic aromatase expression similar to normoxic fish. After 4 weeks of pharmacological treatments, fish were anesthetized, and brain tissues were rapidly excised to measure of aromatase expression and activity.

### RNA Extraction, Reverse Transcription-PCR, Cloning and Sequencing

For RNA extraction, brain tissues were dissected and homogenized using TRI regent (MilliporeSigma), treated with DNase to remove genomic DNA according to manufacturer’s protocol (Promega, Madison, WI, United States) and quantified using a NanoDrop (Thermo Fisher, Waltham, WA, United States). The quality and integrity of total RNA were checked with 1% agarose gel electrophoresis. Reverse transcription (RT) was performed in a 20-μl reaction containing 1 μg of total RNA, 2 pmole of oligo(dT) primer, 10 mM dNTP, 0.1 DTT, 5X first-strand buffer, and 200 units of Superscript III reverse transcriptase (Invitrogen). The reaction mixture was incubated at 42°C for 50 min followed by 70°C for 10 min and stored at –20°C for later use as a template for PCR. For amplifying the partial cDNA fragment of croaker brain aromatase (bAROM), we designed the forward and reversed primers based on highly conserved regions of the known sequences in teleost fishes (Table 1). The PCR conditions were 35 cycles of denaturation at 94°C for 30 s, annealing at 57°C for 30 s and extension at 72^*o*^C for 1 min. The PCR products were electrophoresed on an agarose gel, excised, and purified from the gel, cloned into a pGEM-T easy vector (Promega) and sequenced (DNA Sequencing Facility, Institute for Cellular and Molecular Biology Core Research, University of Texas at Austin, TX, United States). To obtain the full-length cDNA sequence of croaker bAROM, the 5′- and 3′-rapid amplification of cDNA ends (RACE) amplification kit (Invitrogen, Carlsbad, CA, United States) was used following the manufacturer’s protocol using gene-specific primers (Table 1). DNA and amino acid sequences derived from the results of Sanger sequencing were analyzed and compared with the database in GenBank^1^.

**TABLE 1:** Oligonucleotide primers used in this study.

**Primers**	**Sequence**
PSP-F	5′-GGMRTGYATTGGGATGGAAG-3′
PSP-R	5′-CAACASAGTGACCAGRATGG-3′
GSP 5′-1	5′-CCACTATGGCTCTCAGCAGATTGA-3′
GSP 5′-2	5′-CCAGAGTGGGACATCACTGTTGAA-3′
GSP 3′-1	5′-CAATGAGGAAAGCTCTGGAGGATG-3′
GSP 3′-2	5′-ACAGTTCCCAGTCGTTTCTTCCAG-3′
qRT-PCR-F	5′-TCCAGAGGACTGTGGGAATC-3′
qRT-PCR-R	5′-CACCACTATGGCTCTCAGCA-3′
18S-F	5′-AGAAACGGCTACCACATCCA-3′
18S-R	5′-TCCCGAGATCCAACTACGAG-3′

*PSP, partial sequence primer; GSP, gene-specific primer; qRT-PCR, quantitative reverse transcription-polymerase chain reaction; F, forward; R, reverse; M, A/C; R, A/G; Y, C/T; S, C/G.*

### Sequence Alignment and Phylogenetic Analysis

The deduced amino acid sequence of croaker bAROM cDNA was aligned with other vertebrate AROMs (bAROM and ovarian aromatase, oAROM) using MultAlin^2^ sequence alignment according to Corpet (1988).

A phylogenetic tree was generated by the Neighbor-Joining method according to Tamura et al. (2007) and visually depicted by MEGA4 software^3^. The full-length bAROM and oAROM sequences were used in the phylogenetic analysis.

### Quantitative Real-Time PCR Analysis

Gene specific primers (Eurofins MWG Genomics, Louisville, KY, United States) were designed to quantify croaker bAROM mRNA levels (Table 1). NCBI Primer-BLAST tool^4^ and Primer3 software^5^ were used to evaluate the specificity of bAROM and 18S primers. bAROM mRNA levels were determined by a quantitative real-time PCR (qRT-PCR) method using a one-step SYBR Green master mix (Agilent Technologies, La Jolla, CA, United States) as described previously (Rahman and Thomas, 2013). Croaker 18S gene (GenBank accession no.: AY866435) was used as an internal control to determine the relative gene expression. No template control (NTC) was also used to ensure the qRT-PCR amplifications were not the product of extraneous nucleic acid contamination. All reactions were run in duplicate and the cycle threshold (C*t*) values were averaged. The relative mRNA expression results were calculated using the 2^–ΔΔ*Ct*^ method according to Livak and Schmittgen (2001).

### Northern Blot Analysis

Northern blot analysis was performed on total RNA using a DIG RNA-labeling kit according to the manufacturer’s protocol (Roche Diagnostics, Penzberg, Germany). Briefly, total RNA (20 μg) from the hypothalamus was separated on a 1% formaldehyde gel in MOPS buffer (20 mM MOPS, 2 mM sodium acetate, and 1 mM EDTA, pH 7) and blotted onto positive-charged nylon membranes (Turbo Blotter, Schleicher and Schuell Bioscience, Keene, NH, United States). The membranes were then prehybridized in prehybridization buffer at 68°C for 30 min and hybridized with the RNA-labeled probe at 68°C overnight. For negative control, the membrane was hybridized without the RNA-labeled probe. The membranes were then washed with stringency buffer and incubated with DIG-labeled antibody for 30 min. After washes with washing buffer, the membranes were then treated with DIG chemiluminescent solution and exposed to hyperfilm (Amersham Biosciences) to detect the specific bAROM signal.

### Western Blot Analysis

Hypothalamic tissue samples were homogenized with ice-cold HAED buffer (25 mM Hepes, 1 mM EDTA, 10 mM NaCl, 1 mM DTT, and 10 μl/ml of halt protease inhibitor cocktail, pH 7.6). After centrifugation at 10,000 *g* for 15 min, protein concentrations in the supernatant were measured using the Bradford protein assay (Bradford, 1976). Protein extracts (10 μg) were solubilized by boiling in loading buffer, separated on a 10% SDS-PAGE gel, and transferred to a PVDF membrane (Bio-Rad, Hercules, CA, United States). After blocking with 5% non-fat dry milk in TBS-T buffer (50 mM Tris, 100 mM NaCl, 0.1% Tween 20, pH 7.4) for 1 h, the membrane was incubated with anti-aromatase primary antibody (dilution: 1:1,000) or rabbit anti-actin (1:10,000; Southern Biotech, Birmingham, AL, United States) overnight at 4°C. For the peptide block control, the antigen peptide was diluted in blocking buffer (TBS-T containing 0.3% Triton X-100, 5% normal rabbit serum and 1% BSA) containing aromatase antibody (dilution: 1:1,000) and preabsorbed overnight at 4°C. The aromatase peptide antigen was designed based on sequence alignments of known teleost aromatases (Forlano et al., 2001), and the teleost aromatase antibody generated in rabbits were kindly provided by Dr. Andrew H. Bass (Cornell University, Ithaca, NY, United States). Membranes were then washed with TBS-T and incubated with anti-rabbit secondary antibody (1:1,000) for 2 h at room temperature. After washing with TBS-T, membranes were treated with chemiluminescence substrate (Pierce, Rockford, IL, United States) and exposed to hyperfilm (Buckinghamshire, United Kingdom) in the dark. The immunoreactive (IR) intensities of bAROM and action (∼45 kDa protein, used as an internal control) proteins were estimated using ImageJ software (National Institute of Health, Bethesda, MD, United States^6^).

### Radioenzymatic Assay

Aromatase activity was determined in croaker hypothalamus by radioenzymatic assay (REA) using [1β-^3^H]androstenedione (^3^H-A, 250 μCi; PerkinElmer, Waltham, MA, United States) as a tracer and conducted under conditions validated for teleost brain (Gonzalez and Piferrer, 2002). Briefly, hypothalamic tissue samples were homogenized in ice-cold potassium phosphate buffer (100 mM KCl, 10 mM KH_2_PO_4_, 1 mM EDTA, 10 mM DTT, pH 7.4; 1:10, wet weight/vol) and centrifuged at 1,000*g* for min at 4^*o*^C. The crude supernatant fraction (100 μl) was added with 100 μl of cofactor solution containing 100 mM KCl, 10 mM K_2_PO_4_, 1 mM EDTA, 10 mM dithiothreitol, 5 mM glucose-6-phosphate (co-substrate), 1 mM β-nicotinamide adenine dinucleotide phosphate (co-factor), 10 U glucose-6-phosphate dehydrogenase, and 0.6 μM ^3^H-A. The reaction solutions were incubated at 28^*o*^C for 80 min in an incubator. After incubation, the enzymatic reaction was stopped by adding of 500 μl of ice-cold 10% trichloroacetic acid (containing 20 mg charcoal/ml to remove remaining ^3^H-A) and centrifuged at 14,000 *g* for 2 min. The supernatant (200 μl) containing the tritiated water released in the assay was added to 3 ml of scintillation cocktail and vortexed for 5 min at room temperature. The radioactivity was measured with a liquid scintillation counter. Aromatase activity derived from the enzymatic assay was normalized to the total protein present in hypothalamic tissue homogenates, as determined by Bradford protein assay (Bradford, 1976), and expressed as nmol/mg protein/h.

### Double-Labeled Immunohistochemistry

The double-labeled immunofluorescent staining method was used to detect bAROM, NADPH oxidase (NOX), and nNOS expressions in the same teleost brain cells using two unconjugated primary antisera according to Semenova et al. (2014) and Lopez et al. (2017). Briefly, a whole brain sections were deparaffinized in xylene, dehydrated in ethanol dilutions, blocked with blocking solution, and incubated with a mixture of rabbit polyclonal anti-bAROM and goat anti-NOX (dilution: 1:100; Santa Cruz Biotechnology, Dallas, TX, United States) or rabbit polyclonal anti-nNOS (Santa Cruz Biotechnology) and goat anti-NOX antibodies (1:100) overnight at 4^*o*^C. The specificity of anti-bAROM and anti-nNOS antisera were tested previously in immunohistochemical studies in croaker (Rahman and Thomas, 2015) and brains of other teleosts (e.g., rainbow trout, McNeill and Perry, 2006; midshipman, Forlano et al., 2006). Sections were then rinsed with PBS and blocked with blocking solution for 1 h at room temperature. After a rinse with PBS, sections were then incubated with a mixture of Alexa 488-conjugated anti-rabbit (green fluorescence, Invitrogen) and Alexa 594-conjugated anti-goat (red fluorescence, Invitrogen) secondary antibodies (dilution: 1:100) for 1 h at room temperature in the dark. Sections were then rinsed with PBS, rehydrated in ethanol dilutions, mounted in Fluromount-G solution, and the presence of the double-labeled immunofluorescence signal visualized using a confocal microscope (Nikon Eclipse C2, Nikon, Japan).

### Statistical Analysis

All statistical analyses were performed using StatView (SAS Institute Inc., Cary, NC, United States) and GradPad Prism (GraphPad Prism, San Diego, CA, United States) software packages. All experimental data are expressed as arithmetic means ± SEM. Unpaired Student’s *t*-test was used to evaluate the statistical significance for normally distributed data between the two groups. Statistically significant differences between multiple groups were determined using one-way analysis of variance (ANOVA) followed by Fisher’s protected least-significant difference (Fisher’s PLSD) test. For all data analyses, a *p-*value < 0.05 was considered statistically significant.

## Results

### Molecular Characterization, Structural and Phylogenetic Analyses of Croaker bAROM

Using degenerate primers, a partial cDNA fragment (1,000 bp) was amplified from croaker brain RNA by RT-PCR. Sequence analysis showed that this fragment shared high sequence homology with bAROM of other vertebrates. The 5′- and 3′-ends of this partial sequence were amplified by 3′- and 5′-RACE techniques using gene specific primers. The full-length croaker bAROM cDNA is composed of 2226 bp nucleotides which contains 479 bp of 5′-untranslated region (UTR) and 747 bp of 3′-UTR (Supplementary Figure 2) predicting a protein of 502 amino acids with a molecular mass of ∼57 kDa (Figure 1B).

**FIGURE 2:**
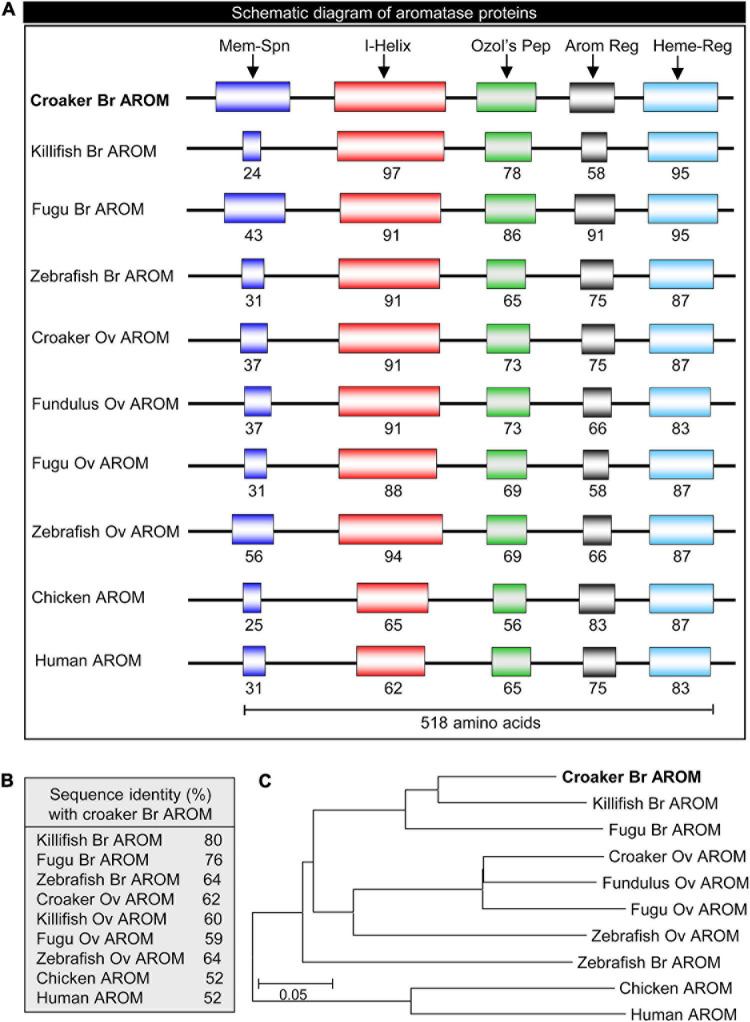
Schematic diagram, sequence identity and phylogeny of croaker brain AROM with the AROM-related proteins of other vertebrates. **(A)** Showing schematic diagram of conserved domains: membrane-spanning domain (Mem-Spn), I-Helix region (I-Helix), Ozol’s peptide region (Ozol’s Pep), aromatic domain (Arom Dom), and heme-binding domain (Heme-B-Dom). See Figure 1 for detailed alignment of amino acid sequences and GenBank accession numbers. **(B)** Percent identities of the deduced amino acid sequence of croaker brain aromatase protein those with the AROM-related proteins of other vertebrates. **(C)** Molecular phylogeny of aromatase proteins. Br, brain; Ov, ovary.

The full-length croaker bAROM cDNA has five major domains/regions, membrane-spanning domain, helix region, Ozol’s peptide region, aromatic domain, and heme-binding domain similar to other vertebrate bAROMs (Figure 1B). These regions are highly conserved within the aligned AROM sequences including the helix region (91–97% identity with other teleost bAROMs, 88–94% teleost oAROMs, and 62–65% with tetrapod AROMs), Ozol’s peptide (65-86% identity with teleost bAROMs, 69–73% with teleost oAROMs, and 56–65% with tetrapod AROMs), aromatic domain (58–91% identity with teleost bAROMs, 58–75% with teleost oAROMs, and 75–83% with tetrapod AROMs) and heme-binding domain (87–95% identity with teleost bAROMs, 83–87% with teleost oAROMs, and 83–87% with tetrapod AROMs) (Figure 2A). The full-length croaker bAROM cDNA displays high sequence identity with bAROM of killifish (80%), Fugu (76%), zebrafish (64%), and relatively low sequence identity with ovarian AROM (oAROM) of zebrafish (64%), croaker (62%), killifish (60%), Fugu (59%), and also tetrapod AROMs (chicken: 52%, human: 52%) (Figure 2B). A phylogenetic analysis was performed to determine the evolutionary relationship of croaker bAROM protein to previously characterized of tetrapod and teleost AROMs. The croaker bAROM is more closely related to the teleost bAROM than the teleost oAROM and tetrapod AROM clades (Figure 2C).

### AROM mRNA Expression in Different Tissues and Discrete Brain Areas

**FIGURE 3:**
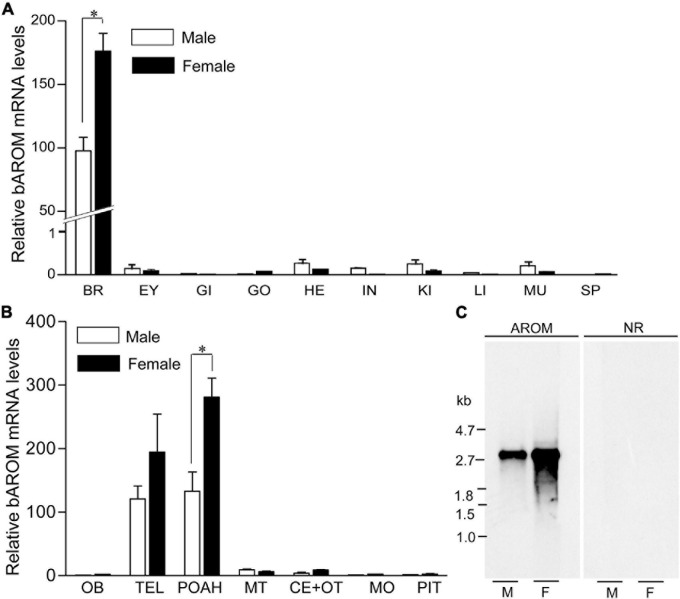
Expression of brain aromatase (bAROM) mRNA in tissues of male and female croaker. **(A)** Total RNA (100 ng) from each tissue was amplified using quantitative real-time PCR (qRT-PCR) in 40 cycles to detect bAROM mRNA. Negative reactions were also used to ensure that the amplification of bAROM mRNA was not from contamination and/or genomic DNA (data not shown). BR, brain; EY, eye; GI, gills; HE, heart; IN, intestine; KI, kidney; LI, liver; MU, muscle; SP, spleen. **(B)** bAROM mRNA levels in different parts of the croaker brain and pituitary by qRT-PCR. The frozen brains were dissected into seven parts: olfactory bulbs (OB), telencephalon (TEL), POAH (preoptic-anterior hypothalamus), midbrain tegmentum (MT), cerebellum plus optic tectum (CE + OT), medulla oblongata (MO), and pituitary (PIT) with aid of a croaker brain atlas (Khan and Thomas, 1993; Rahman and Thomas, 2009). Asterisk indicates significant differences (Student’s *t*-test, *p* < 0.05). **(C)** Northern blot analysis of croaker bAROM transcript in hypothalamic tissues. M, male; F, female.

Quantitative real-time PCR (qRT-PCR) analysis was carried out to determine the expression pattern of croaker bAROM transcript in different tissues and discrete brain areas. qRT-PCR results showed that bAROM mRNA levels were higher in croaker brain tissues (around 97-fold in male and ∼150-fold in female) than in other tissues including eye, gill, testis, ovary, heart, intestine, kidney, liver, muscle, and spleen (Figure 3A). qRT-PCR results also revealed that bAROM mRNA levels were higher in the telencephalon and preoptic-anterior hypothalamic areas compared with other discrete brain regions (e.g., olfactory bulb, midbrain tegmentum, cerebellum plus optic tectum, medulla oblongata) and pituitary gland (Figure 3B).

Northern blot analysis was performed to determine the size and relative abundance of croaker bAROM RNA transcripts. A single hybridization transcript (size: ∼2.8 kb) was detected in croaker hypothalamic tissues. The intensities of the Northern blot bands show than the relative abundance of bAROM mRNA in hypothalamic tissue from females is greater than that in male croaker (Figure 3C).

### Effects of Hypoxia Exposure on bAROM mRNA Levels and Protein Expression

**FIGURE 4:**
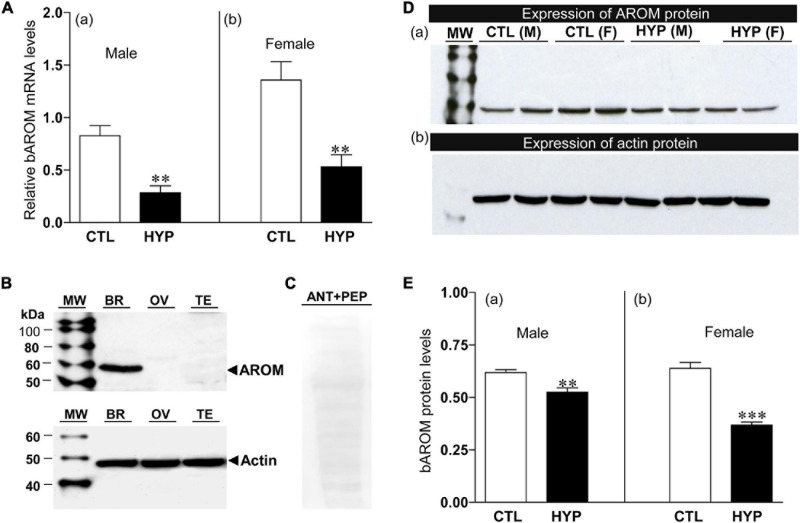
**(A)** Effects of 4 weeks hypoxia (DO: 1.7 mg/L) exposure on brain aromatase (bAROM) mRNA levels in male **(Aa)** and female **(Ab)** croaker hypothalamus determined by qRT-PCR. Here and in the following figures (Figures 5–8), fish were previously exposed to declining DO for an additional 2 days adjustment period, the exposure duration (4 weeks) only refers to the period fish were exposed to target DO (1.7 mg/L). **(B)** bAROM protein expression in croaker tissues. **(C)** Immunoblot reaction blocked by co-incubation of bAROM antibody with specific peptide antigen. kDA, kilodalton; MW, molecular weight marker; BR, brain; OV, ovary; TE, testis; ANT, antibody; PEP, peptide; M, male, F, female. **(D,E)** Effects of 4 weeks hypoxia exposure on bAROM **(Da)** and actin **(Db)** protein expressions and bAROM protein levels [**(Ea)**: male, **(Eb)**: female] in croaker hypothalamus detected by Western blot analysis. Each bar represents mean _ SEM (N = 8). White and black bars represent control (CTL) and hypoxia (HYP). Asterisks indicate significant differences (Student’s t-test, **p < 0.01, ***p < 0.001).

The effects of hypoxia on AROM mRNA levels in croaker hypothalami were determined by qRT-PCR using gene-specific primers. Fish exposed to hypoxia (DO: 1.7 mg/L for 4-week exposure) showed significant decreases in bAROM mRNA levels in hypothalamic tissues in both males and females (Figure 4A). bAROM mRNA levels were decreased in hypothalamic tissues around 2.9-fold (*p* < 0.05, 0.29 ± 0.065) in male and ∼2.5-fold (*p* < 0.05, 0.53 ± 0.11) in female croaker compared to controls (Figures 4Aa,b).

The effects of hypoxia on bAROM protein expression in croaker hypothalami were determined by Western blot analysis. The bAROM antibody detected a major immunoreactive (IR) band around 57 kDa in croaker brains (Figure 4B), which agrees with the predicted molecular mass for croaker bAROM (Figure 1B). No IR signals were detected in croaker testis and ovary. The IR signal in the croaker brain was blocked by preincubation with the bAROM-specific antigen peptide (Figure 4C), confirming the specificity of the immunoreaction with the bAROM antibody. Hypothalamic protein extracts from normoxia- and hypoxia-exposed fish were analyzed for changes in bAROM protein expression. The immunoblot result showed that bAROM protein expression and levels were reduced around 1.18-fold (*p* < 0.01, 0.53 ± 0.02) in male and ∼1.74-fold (*p* < 0.01, 0.37 ± 0.02) in female croaker compared to controls (Figures 4Da,Ea,b). Equal amounts of the protein extracts from hypothalamic tissues were confirmed using actin (size: ∼45 kDa) as a loading control (Figure 4Db).

### Effects of Hypoxia Exposure on AROM Activity and Gonadal Index

**FIGURE 5:**
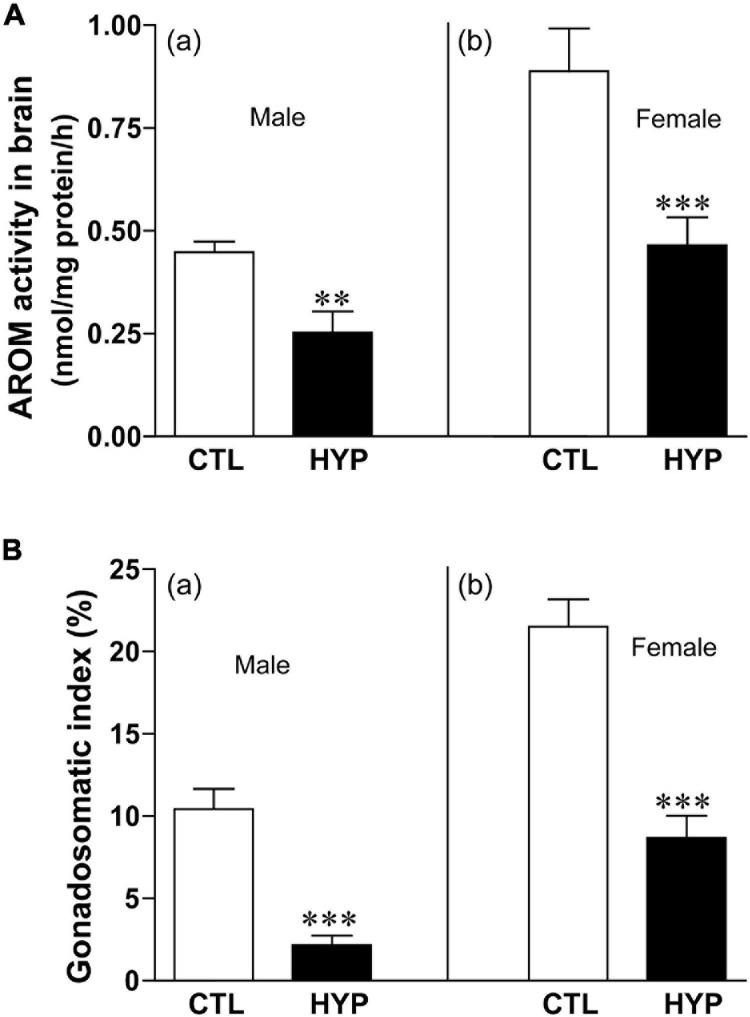
**(A)** Effects of 4 weeks hypoxia (DO: 1.7 mg/L) exposure on aromatase (AROM) activity in male **(Aa)** and female **(Ab)** croaker hypothalamus determined by radioenzymatic assay. **(B)** Effects of 4 weeks hypoxia exposure on gonadosomatic index in male **(Ba)** and female **(Bb)** croaker. Each bar represents mean _ SEM (N = 8–13). White and black bars represent control (CTL) and hypoxia (HYP). Each bar represents mean _ SEM (N = 6–12). White and black bars represent control (CTL) and hypoxia (HYP). Asterisks indicate significant differences (Student’s *t*-test, **p < 0.01, ***p < 0.001).

The effects of hypoxia of AROM activity in croaker hypothalami were determined by radioenzyme assay (REA). Fish exposed to hypoxia (4-weeks) showed a significant decrease in AROM activity in hypothalamic tissues (Figure 5). AROM activity was decreased approximately 1.8-fold (*p* < 0.05, 0.25 ± 0.052) in male croaker exposed to hypoxia compared to that in normoxic controls (Figure 5Aa). A similar trend was also observed in female croaker, where AROM activity was decreased ∼1.91-fold (*p* < 0.05, 0.46 ± 0.068) in hypothalamic tissues from hypoxia-exposed fish compared to normoxia-exposed controls (Figure 5b).

We calculated the gonadosomatic index (GSI = gonad weight/body weight^∗^100) in croaker after hypoxia exposure to determine their stage of gonadal maturity. Both male and female croaker exposed to hypoxia had significantly lower GSI values than the normoxia-exposed controls (Figure 5B). GSI was decreased around 4.8-fold (*p* < 0.05, 2.15 ± 0.59) in male and ∼2.5-fold (*p* < 0.05, 8.68 ± 1.36) in female croaker compared to controls (Figures 5Ba,b).

### Interactive Effects of Hypoxia and NOS or Antioxidant Status on bAROM Expression

**FIGURE 6:**
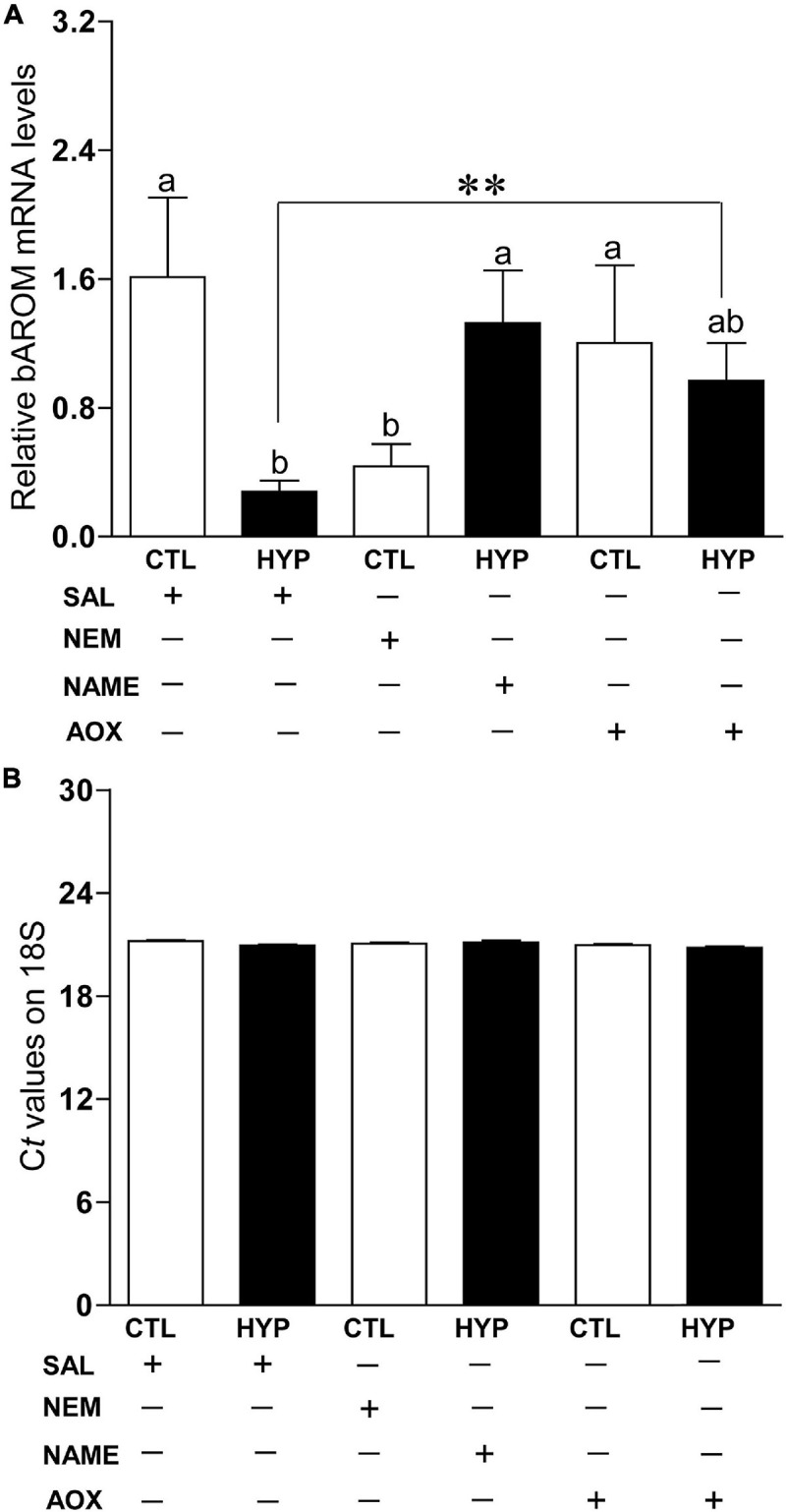
**(A)** Interactive effects of hypoxia (DO: 1.7 mg/L for 4 weeks) and pharmacological drugs on brain aromatase (bAROM) mRNA levels in croaker hypothalamus determined by qRT-PCR. **(B)** C*_*t*_* values of 18S in croaker hypothalamus. Each bar represents mean ± SEM (*N* = 8, results from both sexes were combined, because they were not significant different). White and black bars represent saline (SAL) control (CTL) and hypoxia (HYP). Significant differences (*p* < 0.05) as compared to control (CTL) determined with a multiple range test, Fisher’s PLSD, are indicated with different letters. Asterisks indicate significant differences (Student’s *t*-test, *p < 0.05). *N*-ethylmaleimide (NEM), *N*ω-nitro-L-arginine methyl ester (NAME), antioxidant (AOX, vitamin-E).

To investigate the potential mechanisms of nNOS and AOX status on AROM regulation in teleost brains during hypoxic conditions, we measured bAROM mRNA levels in croaker hypothalamus with or without treatment of NEM, NAME, or AOX after 4 weeks of hypoxia exposure. bAROM mRNA levels were significantly decreased around 3.7-fold (*p* < 0.05, 0.44 ± 0.14) in normoxic fish by treatment with NEM similar to that observed in hypoxia-exposed fish (*p* < 0.05, 0.28 ± 0.07), whereas AROM mRNA levels were dramatically increased in hypoxia-exposed fish by NAME (*p* < 0.05, 1.33 ± 0.32) or AOX (*p* < 0.05, 1.0 ± 0.23) treatments and were similar to those observed in the normoxic saline-injected (1.61 ± 0.49) controls (Figure 6A). There were no significant changes in 18S mRNA levels in croaker hypothalamus with or without treatment of NEM, NAME, or AOX after 4 weeks hypoxia exposure (Figure 6B).

### Interactive Effects of Hypoxia and NOS or Antioxidant Status on AROM Activity

**FIGURE 7:**
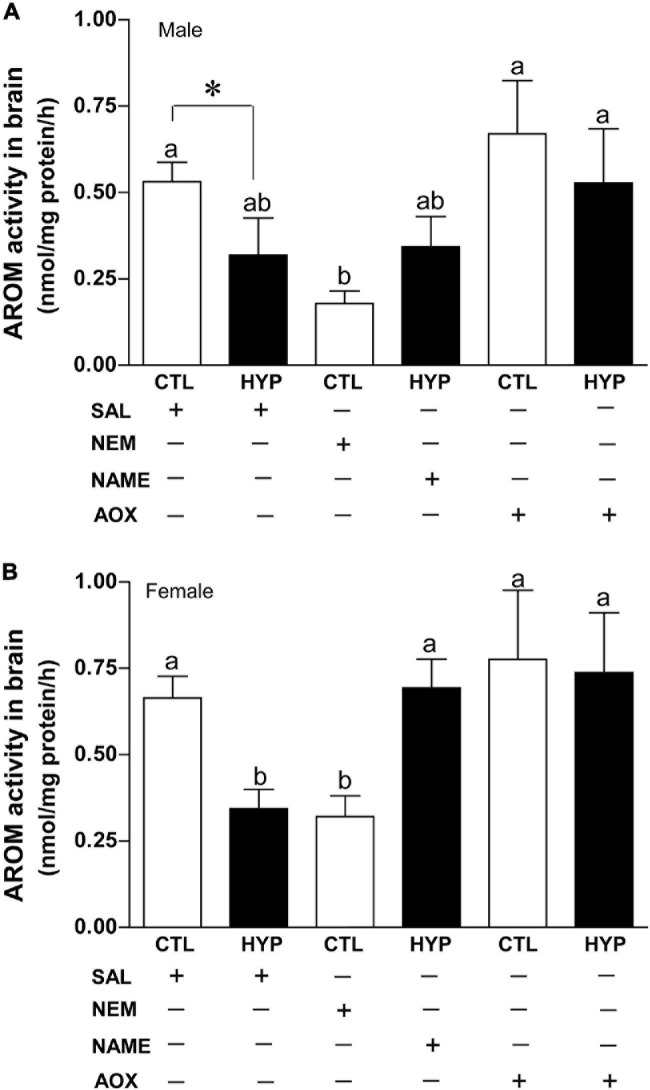
Interactive effects of hypoxia (DO: 1.7 mg/L for 4 weeks) and pharmacological drugs on aromatase (AROM) activity in croaker hypothalamus determined by radioenzymatic assay. AROM activity in male **(A)** and female croaker **(B)**. Each bar represents mean ± SEM (*N* = 5–19). White and black bars represent control (CTL) and hypoxia (HYP). Significant differences (*p* < 0.05) as compared to saline (SAL) control (CTL) determined with a multiple range test, Fisher’s PLSD, are indicated with different letters. Asterisk indicates significant differences (Student’s *t*-test, *p* < 0.05). *N*-ethylmaleimide (NEM), *N*ω-nitro-L-arginine methyl ester (NAME), antioxidant (AOX, vitamin-E).

To explore the potential roles of neuronal nitric oxidase synthase (nNOS) and antioxidant (AOX) status on AROM regulation in croaker brains during hypoxic conditions, we measured AROM activity by REA in croaker hypothalamus with or without treatment with *N*-ethylmaleimide (NEM, an alkylating agent which modifies sulfhydryl groups and increases nNOS expression in croaker hypothalami, Rahman and Thomas, 2015), *N*ω-nitro-L-arginine methyl ester (NAME, a competitive NOS-inhibitor) or vitamin-E (Vit-E, a powerful AOX) after 4 weeks hypoxia exposure (DO: 1.7 mg/L). In male croaker, AROM activity was significantly decreased around 2.9-fold (*p* < 0.05, 0.18 ± 0.036) in normoxic fish by treatment with NEM similar to that observed in hypoxia-exposed fish (*p* < 0.05, 0.32 ± 0.11). Injection with NAME did not significantly alter hypothalamic AROM activity in hypoxic fish, whereas AROM activity was dramatically increased (*p* < 0.05, 0.53 ± 0.16) in hypoxia-exposed fish by AOX treatment similar to that observed in the normoxic saline-injected controls (Figure 7A). A similar trend was also observed in female croaker, where AROM activity was significantly decreased ∼2.1-fold (*p* < 0.05, 0.32 ± 0.06) in normoxic fish by treatment with NEM similar to that observed in hypoxia-exposed fish (*p* < 0.05, 0.34 ± 0.06). AROM activity was dramatically increased in hypoxia-exposed fish by NAME (*p* < 0.05, 0.78 ± 0.2) or AOX (*p* < 0.05, 0.74 ± 0.17) treatment similar to that observed in the normoxic saline-injected (0.66 ± 0.06) controls (Figure 7B).

### Interactive Effects of Hypoxia and NOS or Antioxidant Status on Gonadal Index

**FIGURE 8:**
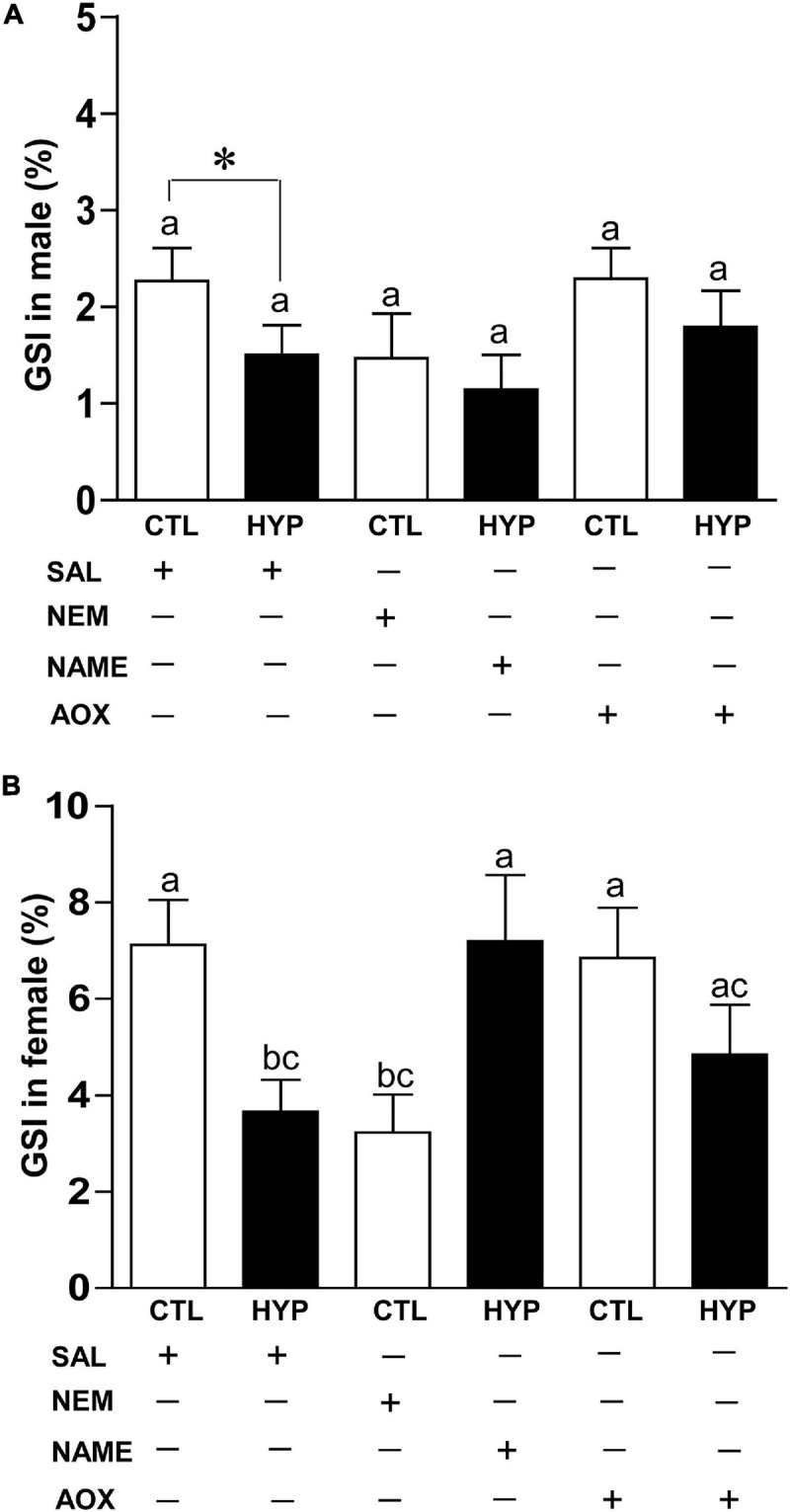
Interactive effects of hypoxia (DO: 1.7 mg/L for 4 weeks) and pharmacological drugs on gonadosomatic index (GSI) in male **(A)** and female **(B)** croaker. Each bar represents mean ± SEM (*N* = 12–22). White and black bars represent control (CTL) and hypoxia (HYP). Significant differences (*p* < 0.05) as compared to saline (SAL) control (CTL) determined with a multiple range test, Fisher’s PLSD, are indicated with different letters. Asterisks indicate significant differences (Student’s *t*-test, *p < 0.05). *N*-ethylmaleimide (NEM), *N*ω-nitro-L-arginine methyl ester (NAME), antioxidant (AOX, vitamin-E).

To investigate their effects on gonadal maturity, we calculated the GSI in croaker gonads with or without treatment with NEM, NAME, or AOX after 4 weeks hypoxia exposure. In male croaker, GSI was significantly decreased around 1.5-fold (*p* < 0.05, 1.5 ± 0.31) in hypoxic fish compared to saline treated controls. Injections with NEM or AOX did not significantly alter the GSI in hypoxia-exposed male fish (Figure 8A). A similar trend was also observed in female croaker, where GSI was significantly decreased around 1.97-fold (*p* < 0.05, 3.65 ± 0.66) in hypoxic saline-injected fish compared with normoxic saline-injected controls (Figure 8B). GSI was also significantly decreased ∼2.2-fold (*p* < 0.05, 3.23 ± 0.79) in normoxic fish by treatment with NEM similar to that observed in hypoxic saline-injected fish, whereas GSI was dramatically increased in hypoxia-exposed fish by NAME (*p* < 0.05, 7.19 ± 1.37) or AOX (*p* < 0.05, 4.84 ± 1.03) treatments similar to that observed in the normoxic saline-injected (7.11 ± 0.93) controls (Figure 8B).

### Immunohistochemical Co-localization of bAROM, NOX, and nNOS Protein Expression

**FIGURE 9:**
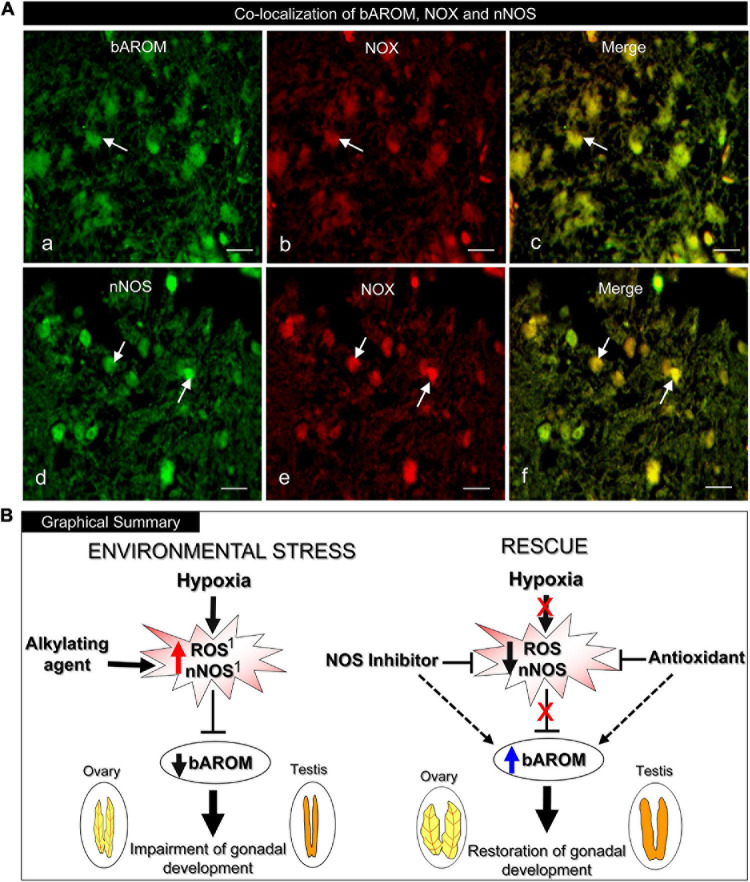
**(A)** Immunohistochemical co-localization of brain aromatase (bAROM), NADPH oxidase (NOX) and neuronal nitric oxide synthase (nNOS) proteins in croaker hypothalamus determined by double-labeled immunofluorescence assay. **(Aa–c)** Arrows indicate neuronal expression of bAROM **(Aa)** and NOX **(Ab)** and **(Ac)** their co-expression (merged). **(Ad–f)** Arrows indicate nNOS **(Ad)** and NOX **(Ae)** and **(Af)** their co-expression (merged). Scale bar = 10 μm. **(B)** Graphical summary of downregulation of bAROM in croaker hypothalamus and impairment of gonadal development after environmental exposure to hypoxia stress and rescue by NOS inhibitor and AOX treatments. The hypoxia-induced increase in reactive oxygen species (ROS) and nNOS resulting in downregulation of bAROM activity is mimicked by treatment with an alkylating agent (*N*-ethylmaleimide, NEM). Treatments with a NOS inhibitor (*N*ω-nitro-L-arginine methyl ester, NAME) and an antioxidant (AOX, vitamin-E) block the hypoxia-induced upregulation of neuronal NOS and ROS which leads to restored AROM activity in brain and subsequently reinstates reproductive functions in gonads. The hypoxia-induced increases of ROS and nNOS in croaker hypothalami and their reversal with NEM and an AOX have been reported previously (^1^Rahman and Thomas, 2015).

To determine the potential anatomical basis for the interactions between bAROM, NOX, and/or nNOS in teleost hypothalami, we examined their co-expression in croaker hypothalami by double-labeled immunofluorescence (DLIF). The DLIF results revealed that AROM and NOX, and nNOS and NOX proteins were co-expressed in cells in the croaker hypothalamus as revealed in the merged images (Figures 9Aa–f).

## Discussion

The present results clearly show that chronic laboratory exposure to hypoxia downregulates bAROM expression and activity in the hypothalamus of a marine teleost model, Atlantic croaker, which is accompanied by impaired gonadal development. These results are consistent with the findings of our earlier field study, which reported reproductive impairment and decreased hypothalamic bAROM mRNA expression in croaker collected from the hypoxic “dead zone” in the northern Gulf of Mexico (Thomas and Rahman, 2012). Exposure to hypoxia in the laboratory and in the dead zone also increased hypothalamic mRNA and protein expression of nNOS and biomarkers of oxidative stress in croaker (Rahman and Thomas, 2017). However, the connection between hypoxia-mediated upregulation of nNOS and downregulation of bAROM had not been explored in the brains of croaker or, to the best of our knowledge, any other vertebrate species. The current results showing the NOS inhibitor, NAME, reverses hypoxia downregulation of bAROM provides clear evidence NOS mediates, at least partly, the modulation of bAROM expression and activity in croaker brains after chronic hypoxia exposure. Hypoxia-induced upregulation of nNOS in croaker hypothalami, in turn is reversed by treatment with an antioxidant, Vit-E, and mimicked by treatment with an alkylating agent, NEM, which also increases superoxide radical production (Rahman and Thomas, 2015). The present results show that these treatments also modulate AROM activity and bAROM mRNA levels, causing opposite responses to those observed with nNOS in croaker hypothalami. Collectively, these studies showing an inverse relationship between nNOS and bAROM after hypoxia and various pharmacological treatments suggests that NOS influences bAROM in croaker hypothalami by decreasing its activity during chronic hypoxia exposure (Figure 9B). Interestingly, the finding that the hypoxia-induced increase in nNOS expression in croaker hypothalami is mimicked by treatment with the aromatase inhibitor, ATD, and reversed by treatment with estradiol-17β (Rahman and Thomas, 2015), suggests that there is a reciprocal relationship between the regulation of these two brain enzymes, and that bAROM also regulates, at least partly, nNOS expression. Clearly, the intimate relationship we have identified between the regulation of these two brain enzymes in the brains of a teleost species exposed to hypoxia is a significant finding that warrants further investigation.

The prediction in the present study that the deduced amino acid sequence of croaker bAROM cDNA has the same five domains/regions (membrane-spanning domain, helix region, Ozol’s peptide region, aromatic domain, and heme-binding domain) as in other teleost brain and ovarian AROM (i.e., bAROM, oAROM) cDNAs, suggests that it has similar catalytic and physiological functions as in other vertebrates. Interestingly, all teleost fish bAROM cDNAs reported so far, including croaker, encode proteins with 488–511 amino acid residues (Halm et al., 2001; Kishida and Callard, 2001; Valle et al., 2002b; Choi et al., 2005; Strobl-Mazzulla et al., 2005; Chaube et al., 2015), whereas fish oAROM cDNAs are larger and predict proteins with 509–523 amino acids (e.g., croaker oAROM cDNA encodes 16 more amino acids than croaker bAROM cDNA) (Gen et al., 2001; Kishida and Callard, 2001; Valle et al., 2002a; Nunez and Applebaum, 2006; Chaube et al., 2015), indicating a major difference between bAROMs and oAROMs in teleost fishes. Phylogenetic analysis revealed that the croaker bAROM isoform is clustered with the bAROM isoform of other teleosts with high similarity, especially with marine teleosts (76–80%), and has lower similarity with oAROM and AROM isoforms of other teleosts (59–64%) and tetrapods (52%), respectively. Notably, both the bAROM and oAROM types of cDNAs in fish are clustered separately from human and chicken AROMs in the phylogenetic tree. Collectively, these results suggest that the teleost bAROM cDNA arose from duplication of the ancestral oAROM gene shared with tetrapods early in the teleost lineage (Kwon et al., 2001). The single croaker bAROM transcript is a similar size, approximately 2.8 kb, to bAROM transcripts in tilapia (∼2.6–2.7 kb, Chang et al., 1997; Kwon et al., 2001), protogynous wrasse bAROM (∼2.6 kb, Choi et al., 2005) and goldfish bAROM (∼3 kb, Gelinas et al., 1998), but larger than in orange-spotted grouper bAROM (∼1.9 kb, Zhang et al., 2004), and shorter than in rainbow trout bAROM (∼3.8 kb, Valle et al., 2002b) and zebrafish bAROM (∼4.4 kb, Kishida and Callard, 2001). In addition, several sizes of bAROM mRNA transcripts have been detected in several teleost fishes by Northern blot analysis which likely represent alternatively spliced forms of the bAROM gene (Halm et al., 2001; Sundaray et al., 2005).

Similar to results in several other teleost fishes (Gonzalez and Piferrer, 2002, 2003; Strobl-Mazzulla et al., 2005; Barney et al., 2008; Dong and Willett, 2008; Chaube et al., 2015), croaker bAROM mRNA is highly expressed in the brain with highest expression in the POAH and TEL regions which regulate reproductive neuroendocrine functions and weakly expressed in the gonads and non-reproductive tissues. It is important to note that similar to female stinging catfish (Chaube et al., 2015), bAROM mRNA levels are higher in hypothalamic tissues of female croaker than in males. Low expression of bAROM mRNA was also observed in the croaker OB, MT, CE + OT, MO, and pituitary but did not show any sex differences. On the other hand, sex differences in bAROM mRNA expression were only detected in one brain region of adult killifish, the OB, with higher expression in females (Dong and Willett, 2008). In marked contrast to findings in the majority of other teleost species, higher AROM activities were detected in the OB, TEL, hypothalamus and pituitary of male than in female seabass and pituitary bAROM mRNA expression was also greater in male South American catfish than in females (Gonzalez and Piferrer, 2003; de Assis et al., 2018). Taken together, these results show that the bAROM gene is highly expressed in brain tissues in fish, ubiquitously found in the entire hypothalamic region, and show sex differences in expression, although these sex differences are observed in different brain regions in the various teleost species examined to date (Garcia-Segura et al., 2003; Piferrer and Blázquez, 2005; Roselli, 2007). bAROM is presumed to be localized in radial glial cells in croaker hypothalami, although this was not confirmed in the present study, because it has been shown to be localized in glial cells in the brains of all the teleost species examined to date (Forlano et al., 2001; Page et al., 2010; Diotel et al., 2011; Xing et al., 2014; Chaube et al., 2015). Glial cell localization of bAROM in croaker hypothalami is also likely because nNOS and NOX, which are localized in the same hypothalamic cells as bAROM in croaker, have been identified in glial cells in other vertebrate models (Kawase et al., 1996; Kim et al., 2008).

In addition to its critical role in reproductive neuroendocrine functions and sexual behavior, there is growing evidence that inhibition of AROM activity causes ovarian masculinization (Guiguen et al., 1999; Thomas and Rahman, 2012) and induces partial sex change in fish (Piferrer et al., 1994; Bhandari et al., 2004; Paul-Prasanth et al., 2013; Goppert et al., 2016), leading to decreases in reproductive fitness. Chronic hypoxia exposure in both field and laboratory settings causes marked impairment of reproductive neuroendocrine functions including downregulation of gonadotropin releasing hormone (GnRH) and gonadotropin (GtH) expression/release and synthesis of the neurotransmitter, serotonin, that controls gonadotropin secretion in croaker (Thomas et al., 2007; Rahman and Thomas, 2009; Thomas and Rahman, 2012), and decreased GnRH and GtH gene expression in zebrafish (Lu et al., 2014) and GtH release in carp (Wang et al., 2008). The present results show that hypoxia exposure also drastically decreases in bAROM mRNA and protein expressions and AROM activity in hypothalamic tissues, which is accompanied by decreases in GSI of both and female croaker, in agreement with our field results from hypoxic sites in the northern Gulf of Mexico (Thomas and Rahman, 2012). In addition, oAROM mRNA levels were decreased in croaker collected from these field sites as well as in a controlled laboratory hypoxia experiment in which ovarian AROM activity was also decreased (Thomas and Rahman, 2012). Similarly, chronic exposure to hypoxia in a laboratory experiment caused a dramatic decline in mRNA levels of both brain and ovarian AROM in zebrafish embryos (Shang et al., 2006), suggesting that the reduction of AROM transcripts is a common phenomenon in both the brain and ovaries of teleost fishes during hypoxia exposure. As expected, these chronic hypoxia-induced decreases in AROM function were accompanied by declines in plasma estradiol-17β levels in teleost fishes, such as carp, croaker, zebrafish, and killifish (Wu et al., 2003; Shang et al., 2006; Thomas et al., 2006, 2007; Landry et al., 2007), and associated in croaker with decreases in hepatic vitellogenin production and yolk accumulation in croaker oocytes resulting in dramatic reductions in fecundity (Thomas et al., 2006, 2007). Moreover, there is growing evidence that inhibition of AROM activity causes ovarian masculinization (Guiguen et al., 1999; Thomas and Rahman, 2012) and induces partial sex change in fish (Piferrer et al., 1994; Goppert et al., 2016), leading to decreases in reproductive fitness. For example, downregulation of AROM in croaker collected from hypoxic field sites in the northern Gulf of Mexico was associated with ovarian masculinization and a male–skewed sex ratio, and similarly, ovarian masculinization (mature sperm in ovaries) also observed in croaker exposed to hypoxia in laboratory studies (Thomas and Rahman, 2012). Thus, hypoxia disruption of the reproductive system in teleosts is both severe and multifaceted.

Exposure to hypoxia also causes increases in free radicals, ROS and RNS, in teleost brains. For example, we have recently demonstrated that hypoxia exposure causes marked increases nNOS, mRNA and protein expressions, and superoxide radical production (e.g., O_2_^∙-^, generated by NADPH oxidase, NOX; Tarafdar and Pula, 2018) in hypothalamic tissues and also increases plasma NOx, a stable oxidized metabolite of nitric oxide (NO), levels in croaker (Rahman and Thomas, 2015, 2017). Similarly, McNeill and Perry (2006) demonstrated that short-term exposure to hypoxia caused increases nNOS mRNA levels in rainbow trout brain, which was accompanied by increases in plasma NOx levels. These studies and the present findings suggest that hypoxia causes excessive production of ROS and/or RNS through an increase in nNOS and/or NOX activity resulting in increased oxidative/nitrative stress. The available evidence suggests that downregulation of bAROM and subsequent reproductive impairment during hypoxia exposure is mediated by this increased RNS/ROS-induced stress (e.g., O_2_^∙-^) through activation NOS and/or NOX. We have recently demonstrated hypoxia-induced upregulation of nNOS mRNA and protein expression of O_2_^∙-^ production in croaker hypothalami is reduced by the NOS-inhibitor, NAME, and an antioxidant (AOX, Vit-E; Rahman and Thomas, 2015). Moreover, the alkylating agent, NEM, a chemical which covalently modifies sulfhydryl groups (Smyth et al., 1964), increases nNOS mRNA and protein expression, and O_2_^∙-^ production in croaker hypothalamus under normoxic conditions, similar to the changes observed under hypoxic conditions (Rahman and Thomas, 2015). The present results show that hypoxia and these pharmacological treatments that modulate nNOS activity and RNS/RAO production also alter bAROM expression and activity. Administration of AOX or the NOS-inhibitor, NAME, fully restored AROM activity and bAROM mRNA levels in croaker hypothalamus, and partially/fully reinstated gonadal fitness (i.e., GSI), which clearly implicates NO and O_2_^∙-^ generation in hypoxia-induced downregulation of bAROM. The double-labeled immunofluorescence results showing the presence of bAROM, nNOS, and NOX proteins in the same hypothalamic cells in croaker provides a cellular context for their interactions. However, changes in bAROM expression and activity in croaker hypothalami induced by hypoxia and the pharmacological treatments may be partially mediated indirectly through their effects on ovarian AROM activity and estradiol-17β production. The expression and activity of bAROM in the brain of several teleosts species is modulated by circulating estradiol-17β levels and ovarian AROM activity (Page et al., 2010; Diotel et al., 2011). Both oAROM expression/activity and plasma estradiol-17β levels were decreased in croaker after chronic hypoxia exposure (Thomas et al., 2007; Thomas and Rahman, 2012). Croaker were treated with NAME, NEM and an AOX systemically in the present study so they likely exerted similar effects on oAROM as we observed with bAROM. This is supported by a report that an oxidizing agent, phenol, drastically increased H_2_O_2_, a potent ROS, and hydroxyl radical (OH∙, a highly reactive ROS) and reduced AROM activity and oAROM mRNA levels in carp ovaries (Das et al., 2016). Treatment of mammalian models with NEM and NO-donors, *S*-nitro-*N*-acetylpenicillamine (SNAP) and NOC18, decrease AROM activity and estradiol-17β production by ovarian follicle cells (Snyder et al., 1996; Masuda et al., 2001), and AOX treatment restored AROM activity in placental microsomes after oxidant stress (Milczarek et al., 2008). Thus, downregulation of bAROM activity and neuroendocrine function in croaker after hypoxia exposure is potentially mediated by its oxidative/nitrative effects in the ovary on oAROM as well as direct effects in the hypothalamus.

## Conclusion

The results demonstrate that hypoxia reduces AROM activity and bAROM mRNA and protein expressions in the hypothalamus of a hypoxia-tolerant marine teleost, Atlantic croaker. Notably, the present results also provide clear evidence that the hypoxia-mediated downregulation of bAROM is partially mediated through NOS and alterations in the antioxidant status. The results show for the first time that during hypoxic stress, administration of a NOS-inhibitor or AOX (Vit-E), leads to increased bAROM transcript expression and AROM activity in the hypothalamus and restores gonadal development. An increased understanding of the mechanism of downregulation of hypothalamic bAROM during exposure of teleost fish to environmental hypoxia has major physiological implications because the enzyme performs critical functions in the neuroendocrine control of reproduction, maintenance of sexual plasticity, and neuroprotection. This knowledge is also of broad ecological significance because environmental hypoxia is widespread in coastal regions and has recently dramatically increased worldwide due to increased eutrophication as a result of human activities. Although NOS inhibitor treatments and the AOX status clearly influence bAROM activity and both nNOS and NOX are co-localized with bAROM in the croaker hypothalamus, additional research will be required to determine whether they exert their AROM effects solely in this tissue, or whether these pharmacological treatments also exert their effects indirectly, for example, by their actions on AROM in the ovaries to restore estradiol-17β production resulting in increases in bAROM activity. Further research on the mechanisms of bAROM downregulation during hypoxia exposure will be required to answer this question.

## Data Availability Statement

All relevant data of this manuscript are available from the corresponding author upon request.

## Ethics Statement

All animal husbandry practices and laboratory experimental procedures were approved by the University of Texas at Austin Institutional Animal Care and Use Committee (IACUC, protocol #09022701). Fish were also handled and/or cared according to the Guide for Care and Use Animals for research in the United States National Research Council Committee (https://grants.nih.gov/grants/olaw/guide-for-the-care-and-use-of-laboratory-animals.pdf).

## Author Contributions

MSR and PT designed the research. MSR performed the research, analyzed the data, and wrote the manuscript. Both authors revised the manuscript and approved to submit the final version of manuscript.

## Conflict of Interest

The authors declare that the research was conducted in the absence of any commercial or financial relationships that could be construed as a potential conflict of interest.

## Publisher’s Note

All claims expressed in this article are solely those of the authors and do not necessarily represent those of their affiliated organizations, or those of the publisher, the editors and the reviewers. Any product that may be evaluated in this article, or claim that may be made by its manufacturer, is not guaranteed or endorsed by the publisher.

## Footnotes

1
http://www.ncbi.nlm.nih.gov/genbank

2
http://multalin.toulouse.inra.fr/multalin

3
http://megasoftware.net

4
http://www.ncbi.nlm.nih.gov/tools/primer-blast

5
http://bioinfo.ut.ee/primer3-0.4.0

6
http://rsb.info.nih.gov/ij/

## Abbreviations

AROM: cytochrome P450 aromatase
bAROM: brain-type aromatase (Cyp19a1b)
oAROM: ovarian-type aromatase (Cyp19a1a)
NOS: nitric oxide synthase
ROS: reactive oxygen species
RNS: reactive nitrogen species
NEM: *N*-ethylmaleimide
AOX: antioxidant
NAME: *N* ω -nitro-L-arginine methyl ester
RT-PCR: reverse-transcription polymerase chain reaction
RACE: rapid amplification of cDNA ends
GSP: gene specific primer
UTR: untranslated region
ORF: open reading frame
DO: dissolved oxygen
POAH: preoptic-anterior hypothalamus
PIT: pituitary
OB: olfactory bulb
MT: mid-brain tegmentum
TEL: telencephalon.
