# Supplementary material for: Molecular Characterization and Expression of Cytochrome P450 Aromatase in Atlantic Croaker Brain: Regulation by Antioxidant Status and Nitric Oxide Synthase During Hypoxia Stress
Source: Front Physiol. 2021 Aug 9;12:720200. doi: 10.3389/fphys.2021.720200 (PMC8381199; doi:10.3389/fphys.2021.720200)
Supplement: Supplementary file 1 [file Presentation_1.PPT]

## Slide 1
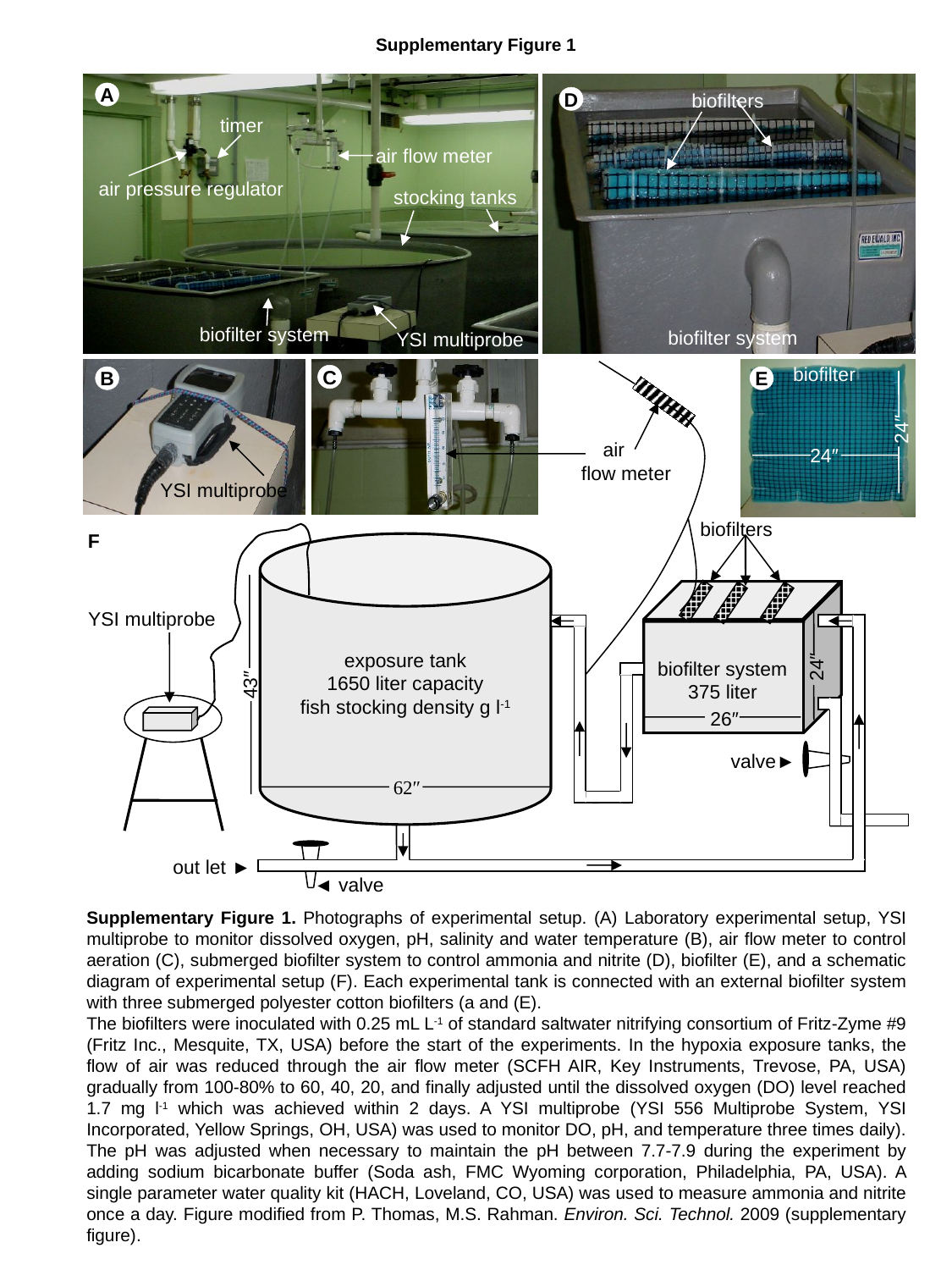

Supplementary Figure 1
biofilters
A
D
timer
air flow meter
air pressure regulator
stocking tanks
biofilter system
biofilter system
YSI multiprobe
biofilter
C
B
E
24″
 air
flow meter
24″
YSI multiprobe
biofilters
F
YSI multiprobe
exposure tank
1650 liter capacity
fish stocking density g l-1
24″
biofilter system
375 liter
43″
26″
valve►
62″
out let ►
◄ valve
Supplementary Figure 1. Photographs of experimental setup. (A) Laboratory experimental setup, YSI multiprobe to monitor dissolved oxygen, pH, salinity and water temperature (B), air flow meter to control aeration (C), submerged biofilter system to control ammonia and nitrite (D), biofilter (E), and a schematic diagram of experimental setup (F). Each experimental tank is connected with an external biofilter system with three submerged polyester cotton biofilters (a and (E).
The biofilters were inoculated with 0.25 mL L-1 of standard saltwater nitrifying consortium of Fritz-Zyme #9 (Fritz Inc., Mesquite, TX, USA) before the start of the experiments. In the hypoxia exposure tanks, the flow of air was reduced through the air flow meter (SCFH AIR, Key Instruments, Trevose, PA, USA) gradually from 100-80% to 60, 40, 20, and finally adjusted until the dissolved oxygen (DO) level reached 1.7 mg l-1 which was achieved within 2 days. A YSI multiprobe (YSI 556 Multiprobe System, YSI Incorporated, Yellow Springs, OH, USA) was used to monitor DO, pH, and temperature three times daily). The pH was adjusted when necessary to maintain the pH between 7.7-7.9 during the experiment by adding sodium bicarbonate buffer (Soda ash, FMC Wyoming corporation, Philadelphia, PA, USA). A single parameter water quality kit (HACH, Loveland, CO, USA) was used to measure ammonia and nitrite once a day. Figure modified from P. Thomas, M.S. Rahman. Environ. Sci. Technol. 2009 (supplementary figure).
