# Supplementary material for: Molecular Characterization and Expression of Cytochrome P450 Aromatase in Atlantic Croaker Brain: Regulation by Antioxidant Status and Nitric Oxide Synthase During Hypoxia Stress
Source: Front Physiol. 2021 Aug 9;12:720200. doi: 10.3389/fphys.2021.720200 (PMC8381199; doi:10.3389/fphys.2021.720200)
Supplement: Supplementary file 2 [file Presentation_2.PPT]

## Slide 1
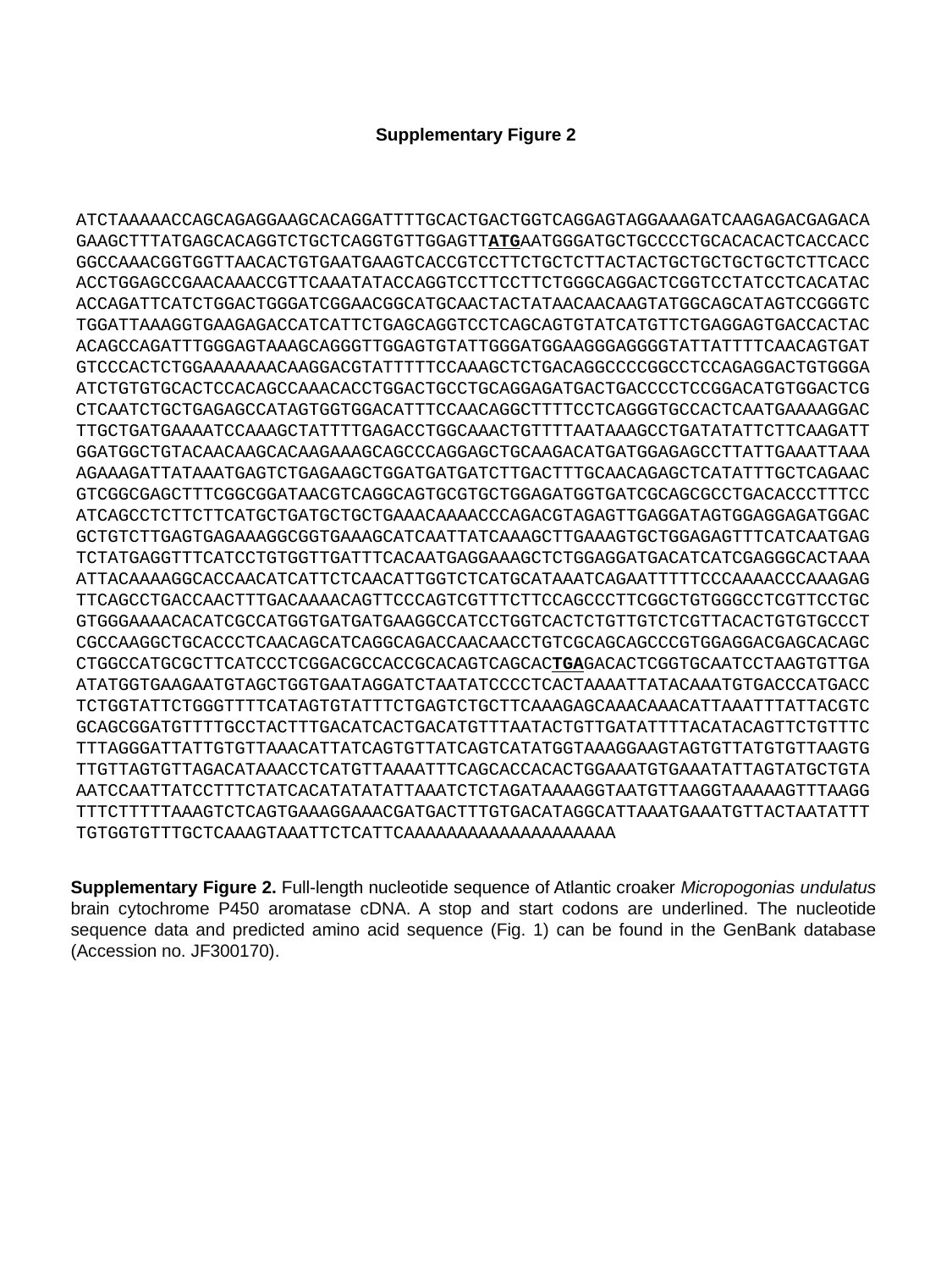

Supplementary Figure 2
ATCTAAAAACCAGCAGAGGAAGCACAGGATTTTGCACTGACTGGTCAGGAGTAGGAAAGATCAAGAGACGAGACAGAAGCTTTATGAGCACAGGTCTGCTCAGGTGTTGGAGTTATGAATGGGATGCTGCCCCTGCACACACTCACCACCGGCCAAACGGTGGTTAACACTGTGAATGAAGTCACCGTCCTTCTGCTCTTACTACTGCTGCTGCTGCTCTTCACCACCTGGAGCCGAACAAACCGTTCAAATATACCAGGTCCTTCCTTCTGGGCAGGACTCGGTCCTATCCTCACATACACCAGATTCATCTGGACTGGGATCGGAACGGCATGCAACTACTATAACAACAAGTATGGCAGCATAGTCCGGGTCTGGATTAAAGGTGAAGAGACCATCATTCTGAGCAGGTCCTCAGCAGTGTATCATGTTCTGAGGAGTGACCACTACACAGCCAGATTTGGGAGTAAAGCAGGGTTGGAGTGTATTGGGATGGAAGGGAGGGGTATTATTTTCAACAGTGATGTCCCACTCTGGAAAAAAACAAGGACGTATTTTTCCAAAGCTCTGACAGGCCCCGGCCTCCAGAGGACTGTGGGAATCTGTGTGCACTCCACAGCCAAACACCTGGACTGCCTGCAGGAGATGACTGACCCCTCCGGACATGTGGACTCGCTCAATCTGCTGAGAGCCATAGTGGTGGACATTTCCAACAGGCTTTTCCTCAGGGTGCCACTCAATGAAAAGGACTTGCTGATGAAAATCCAAAGCTATTTTGAGACCTGGCAAACTGTTTTAATAAAGCCTGATATATTCTTCAAGATTGGATGGCTGTACAACAAGCACAAGAAAGCAGCCCAGGAGCTGCAAGACATGATGGAGAGCCTTATTGAAATTAAAAGAAAGATTATAAATGAGTCTGAGAAGCTGGATGATGATCTTGACTTTGCAACAGAGCTCATATTTGCTCAGAACGTCGGCGAGCTTTCGGCGGATAACGTCAGGCAGTGCGTGCTGGAGATGGTGATCGCAGCGCCTGACACCCTTTCCATCAGCCTCTTCTTCATGCTGATGCTGCTGAAACAAAACCCAGACGTAGAGTTGAGGATAGTGGAGGAGATGGACGCTGTCTTGAGTGAGAAAGGCGGTGAAAGCATCAATTATCAAAGCTTGAAAGTGCTGGAGAGTTTCATCAATGAGTCTATGAGGTTTCATCCTGTGGTTGATTTCACAATGAGGAAAGCTCTGGAGGATGACATCATCGAGGGCACTAAAATTACAAAAGGCACCAACATCATTCTCAACATTGGTCTCATGCATAAATCAGAATTTTTCCCAAAACCCAAAGAGTTCAGCCTGACCAACTTTGACAAAACAGTTCCCAGTCGTTTCTTCCAGCCCTTCGGCTGTGGGCCTCGTTCCTGCGTGGGAAAACACATCGCCATGGTGATGATGAAGGCCATCCTGGTCACTCTGTTGTCTCGTTACACTGTGTGCCCTCGCCAAGGCTGCACCCTCAACAGCATCAGGCAGACCAACAACCTGTCGCAGCAGCCCGTGGAGGACGAGCACAGCCTGGCCATGCGCTTCATCCCTCGGACGCCACCGCACAGTCAGCACTGAGACACTCGGTGCAATCCTAAGTGTTGAATATGGTGAAGAATGTAGCTGGTGAATAGGATCTAATATCCCCTCACTAAAATTATACAAATGTGACCCATGACCTCTGGTATTCTGGGTTTTCATAGTGTATTTCTGAGTCTGCTTCAAAGAGCAAACAAACATTAAATTTATTACGTCGCAGCGGATGTTTTGCCTACTTTGACATCACTGACATGTTTAATACTGTTGATATTTTACATACAGTTCTGTTTCTTTAGGGATTATTGTGTTAAACATTATCAGTGTTATCAGTCATATGGTAAAGGAAGTAGTGTTATGTGTTAAGTGTTGTTAGTGTTAGACATAAACCTCATGTTAAAATTTCAGCACCACACTGGAAATGTGAAATATTAGTATGCTGTAAATCCAATTATCCTTTCTATCACATATATATTAAATCTCTAGATAAAAGGTAATGTTAAGGTAAAAAGTTTAAGGTTTCTTTTTAAAGTCTCAGTGAAAGGAAACGATGACTTTGTGACATAGGCATTAAATGAAATGTTACTAATATTTTGTGGTGTTTGCTCAAAGTAAATTCTCATTCAAAAAAAAAAAAAAAAAAAA
Supplementary Figure 2. Full-length nucleotide sequence of Atlantic croaker Micropogonias undulatus brain cytochrome P450 aromatase cDNA. A stop and start codons are underlined. The nucleotide sequence data and predicted amino acid sequence (Fig. 1) can be found in the GenBank database (Accession no. JF300170).
